# Impact of hyperglycemia on morbidity and mortality, length of hospitalization and rates of re-hospitalization in a general hospital setting in Brazil

**DOI:** 10.1186/1758-5996-2-49

**Published:** 2010-07-21

**Authors:** Silmara AO Leite, Simone B Locatelli, Sabrina P Niece, Aline RF Oliveira, Deborah Tockus, Thaísa Tosin

**Affiliations:** 1 Internal Medicine of Universidade Positivo, Curitiba, PR, Brasil; 2 Universidade Positivo, Curitiba, PR, Brasil

## Abstract

**Background:**

Hyperglycemia in hospitalized patients is known to be related to a higher incidence of clinical and surgical complications and poorer outcomes. Adequate glycemic control and earlier diagnosis of type 2 diabetes during hospitalization are cost-effective measures.

**Methods:**

This prospective cohort study was designed to determine the impact of hyperglycemia on morbidity and mortality in a general hospital setting during a 3-month period by reviewing patients' records. The primary purposes of this trial were to verify that hyperglycemia was diagnosed properly and sufficiently early and that it was managed during the hospital stay; we also aimed to evaluate the relationship between in-hospital hyperglycemia control and outcomes such as complications during the hospital stay, extent of hospitalization, frequency of re-hospitalization, death rates and number of days in the ICU (Intensive Care Unit) after admission. Statistical analyses utilized the Kruskall-Wallis complemented by the "a posteriori" d.m.s. test, Spearman correlation and Chi-squared test, with a level of significance of 5% (p < 0.05).

**Results:**

We reviewed 779 patient records that fulfilled inclusion criteria. The patients were divided into 5 groups: group (1) diabetic with normal glycemic levels according to American Diabetes Association criteria for in-hospital patients (n = 123); group (2) diabetics with hyperglycemia (n = 76); group (3) non-diabetics with hyperglycemia (n = 225); group (4)diabetics and non-diabetics with persistent hyperglycemia during 3 consecutive days (n = 57) and group (5) those with normal glucose control (n = 298). Compared to patients in groups 1 and 5, patients in groups 2, 3 and 4 had significantly higher mortality rates (17.7% vs. 2.8%) and Intensive Care Unit admissions with complications (23.3% vs. 4.5%). Patients in group 4 had the longest hospitalizations (mean 15.5 days), and group 5 had the lowest re-hospitalization rate (mean of 1.28 hospitalizations). Only 184 (51.4%) hyperglycemic patients had received treatment. An insulin "sliding-scale" alone was the most frequent treatment used, and there was a wide variation in glucose target medical prescriptions. Intra Venous insulin infusion was used in 3.8% of patients in the ICU. Glycohemoglobin(A1C) was measured in 11 patients(2.2%).

**Conclusions:**

Hospital hyperglycemia was correlated with, among other parameters, morbidity/mortality, length of hospitalization and number of re-hospitalizations. Most patients did not have their glycemic levels measured at the hospital; despite the high number of hyperglycemic patients not diagnosed as diabetics, A1C was not frequently measured. Even when patients are assessed for hyperglycemia, they were not treated properly.

## Introduction

Hyperglycemia in a hospital, with or without a previous history of diabetes, setting occurs frequently in patients with acute myocardial infarction, trauma, burns, cardiac surgery, stroke and septicemia [1,2].

Most observational and retrospective studies have reported that hyperglycemia in patients with severe disease is associated with an increased risk of complications, longer ICU stay and higher mortality rates [3-6].

The pathophysiological mechanism that might explain the relationship between increased glycemia and likelihood to develop complications or death in critically ill patients has not been established. It is controversial whether the increase in glycemic levels is independently associated with a worse prognosis or whether it may indicate a more severe disease with a stronger response to stress [7].

Several randomized prospective studies have shown that intense control of glycemia reduces multiple organ failure, infection, mortality both in the short and long term, and hospitalization and ICU stays, with consequent lower total hospitalization costs [7-9].

Trials examining the effects of tighter glucose control have had conflicting results. Systematic reviews and meta-analyses have also had differing conclusions [10,11].

The purpose of this study was to assess how glycemic levels are managed in hospitalized patients and the impact of hyperglycemia on patient outcomes both in the ward and the ICU of a general hospital.

## Methods

A prospective cohort study was conducted with the evaluation of all in-patients from the Hospital Cruz Vermelha Brasileira - Filial do Paraná, admitted between September 4, 2007 and December 4, 2007. The patients were followed until discharge. The patients studied were in both private and public health systems, from wards, private rooms and from the general and coronary ICUs.

Items evaluated were: patient age and gender, use of drugs for diabetes treatment, use of medications that might alter glycemic levels, whether there had been a previous diabetes diagnosis, reason for hospitalization and length of hospital stay, concurrent clinical and surgical conditions, glycated hemoglobin, plasmatic and capillary glycemia, endocrinologist evaluation, need for re-hospitalization, and the definition of cases who were given IV insulin infusion in the ICU.

Exclusion criteria were: hospital stay shorter than 24 hours, patients who were under 18 years of age and pregnant women.

The data were obtained by accessing electronic patient records, in addition to reading the printed and written patient records available in files at the nursing stations of each hospital section. Whenever a record was incomplete, additional information was requested directly from the patient or family members, and they were asked to sign the Informed Consent Form.

We did not continue to follow the patients who had not had any glucose tests within 72 hours of admission. Patients in the general and coronary ICUs were followed even after discharge from the ICU but were still in the hospital. We used the reference values for the exams established by the *American Diabetes Association *(ADA) [12]. In-hospital hyperglycemia in the wards was defined as fasting plasma glucose levels higher than 110 mg/dL and/or capillary glucose levels higher than 180 mg/dL after eating and higher than 130 mg/dL before meals; hyperglycemia in the ICU was defined as glucose levels higher than 110 mg/dL; in general, hyperglycemia was defined as higher than 180 mg/dL at both locations. Persistent hyperglycemia was defined as glucose levels higher than 180 mg/dL one or more times during three days in the hospital[14]. Controlled glycemia was defined as no persistent hyperglycemia during the patient's stay either in the ward or the ICU. Hypoglycemia was defined as glucose levels lower than 60 mg/dL.

Plasma glucose and glycated hemoglobin tests were performed by the hospital's laboratory. Plasma glucose was measured using the Enzymatic/Colorimetric Method with computerized equipment and a kit from BioTécnica Indústria e Comércio Ltda. The glycated hemoglobin test was performed by determining blood glucohemoglobin levels using the cation exchange method with the Glico-Teck^® ^kit (Katal Biotecnológica Indústria e Comércio Ltda). Capillary glucose measurements were entered into the printed patient records after having been checked by the nursing team with the *Accu-Check Advantage*^® ^device (Roche). This device satisfied the requirements of rule 98/79/CE regarding products for diagnosis *in vitro*.

The data were collected daily by five 4th-year Universidade Positivo medical school students, who were trained by the endocrinologist and diabetes specialist. Prior to the study, a two-week pilot project was conducted to ensure the applicability of the chosen methodology and the correct formulation of the data collection protocols.

This study was approved by the Committee of University Research Ethics of Universidade Positivo and by the higher management of the Hospital.

Microsoft Excel was used to enter patient records, and the statistical analysis was performed with Statistic version 6.0. First, patients were categorized in groups based on capillary and lab glucose levels. The groups were compared using the Kruskal-Wallis test. The normal distribution assumption and the equality of variance were tested using the Levene's test. To verify the significant differences among the groups, the "a posteriori" d.m.s. test was used to supplement the Kruskal-Wallis test. The Spearman's coefficient and Chi-squared test were used to evaluate the association among some of the variables. The significance level used in the comparisons was 5%.

## Results

Between September and December 2007, 2140 patients were admitted to the Hospital Cruz Vermelha Brasileira - Filial do Paraná, a general hospital where most attending physicians are general practitioners.

**Figure 1:**
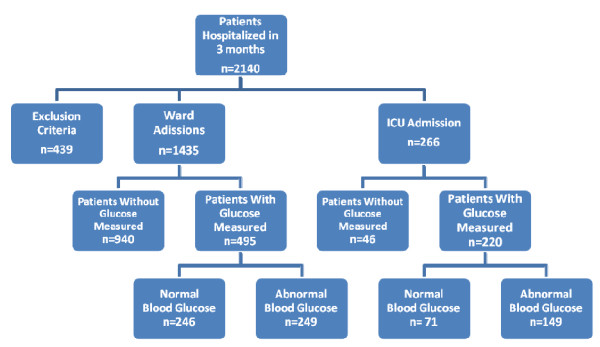
**Total number of patients in-hospital during 3 months and inclusion algorithm in the study (Glucose is expressed in mg/dL)**.

We evaluated patients hospitalized both through the SUS (Public Brazilian Health Care System) and the private health care system. Half of the beds were assigned for surgery and the other half for clinical treatment. The algorithm for patient inclusion in this study is shown in Figure 1. A total of 719 patients had their glucose levels assessed at the time of hospital admission.

Patients whose glucose levels were tested during their stay or upon admission and/or during hospitalization in the ICU and/or ward (n = 779) were included in the analysis. These patients were categorized into five groups according to their hospital hyperglycemia status. Table 1 shows the patients' demographic information. There were similar numbers of men and women in the study.

**Table 1:** Patient demographics according to glycemic status:

Groups	Number of Patients	Mean Ages	SD	%
Group (1)	123	64.7	14.1	15.8

Group (2)	76	63.9	14.4	9.8

Group (3)	225	59.1	19.4	28.9

Group (4)	57	64.1	15.1	7.3

Group (5)	298	53.8	17.3	38.3

TOTAL	779	58.8	17.6	100

Group(1) = DM+ Normal Glucose; Group(2) = DM+ Hyperglycemia; Group(3) = Hospital Hyperglycemia; Group(4) = Persistent Hyperglycemia; Group(5) = Normal Glycemia

Although 36.2% of the patients (n = 225) had in-hospital hyperglycemia without a previous diabetes diagnosis, the A1C test was administered in only 2.2% of the patients (n = 11).

Patients with hyperglycemia had significantly longer hospital stays, regardless of diabetes (Table 2).

**Table 2:** Length of hospital stay for different patient groups

Glucose levels and diagnosis (groups)	Average length of hospitalizations (days)	Average number of hospitalizations
Group (1)	6.3	1.5

Group (2)	8.7	1.5

Group (3)	10.1	1.6

Group (4)	15.5	1.5

Group (5)	6.3	1.3

Group(1) = DM+ Normal Glucose; Group(2) = DM+ Hyperglycemia; Group(3) = Hospital Hyperglycemia; Group(4) = Persistent Hyperglycemia; Group(5) = Normal Glycemia

Diabetes Mellitus (DM) was the cause of admission for 16 cases, 12 of whom were admitted into the ward and 4 into the ICU; admission for hypoglycemia occurred for only 1 case. Hypoglycemic events occurred in 18 patients in the ward and 36 patients in the ICU.

Of the 249 patients who developed hyperglycemia in the ward, only 106 were treated. Of the treated patients, 34 patients remained on oral agents. Insulin was prescribed to 70% of the patients (n = 74), 75% of whom (n = 54) were treated with sliding-scale insulin alone; 18 patients were treated with basal insulin NPH associated with sliding-scale regular insulin. In most cases, hyperglycemia was treated when glucose levels were higher than 200 mg/dl.

Medication that interfered in glucose metabolism, such as corticoid therapy, was used in 49 patients, who then became hyperglycemic; NPH insulin was given to only 14 of these patients.

In the ICU, patients in groups 2, 3 and 4 (n= 149) had a significantly higher mortality rate of 16.1% (n = 24) compared to the 2.8% (n = 2) in patients with normal glucose (n = 71) (p = 0.0001) (Table 3).

**Table 3:** Mortality in patients with normal and abnormal glucose levels: Ward patients vs. ICU patients

	Ward Patients		ICU Patients	
**Glucose Level**	**Normal**	**Abnormal**	**Normal**	**Abnormal**

**Number of Patients**	**246**	**249**	**71**	**149**

**Number of Deaths**	**0**	**4**	**2**	**24**

**Mortality Rate**	**0%**	**1.6%**	**2.8%**	**16.1%**

Patients with hyperglycemia in the ward (n = 249) were, significantly more likely to require admission into the ICU 23.3% (n = 57) compared to 4.5% (n = 11) of those who were normoglycemic (n = 246) (p = 0.0001)).

In the ICU, 78 of the 149 patients with glucose levels higher than 180 mg/dL were treated; the sliding scale insulin alone was used in 86% of the cases. Continuous IV insulin infusion was administered to 3.8% of the patients.

There is no clear algorithm for making temporary corrective changes to reach and maintain the recommended blood glucose levels successfully within a pre-specified target range of glucose levels.

Although a prevalence of in-hospital hyperglycemia was shown, an endocrinology evaluation was required in only 4.8% of the cases.

## Discussion

In this cohort of patients hospitalized in a community hospital setting, we demonstrated that hyperglycemia led to poorer clinical outcomes, with increased morbidity and mortality and length of hospital stay. Similar results had been reported in other studies [1,13,19,21].

Most patients were not checked for glucose levels during their stay in the hospital, which shows that the glucose level parameter is not adequately valued as an impacting factor in the hospital setting.

Because half of the patients were admitted for surgical reasons and their glucose levels may not have been tested prior to admission, the number of patients whose glucose levels were not evaluated may have been overestimated.

It is noteworthy that hyperglycemia was not treated in 57.5% of the patients, probably because treatment was started only when blood glucose levels were higher than 200 mg/dL, although the recommendations for managing inpatient hyperglycemia are to target random blood glucose levels lower than 180 mg/dL [22].

In our evaluation of patients in the ward, among those whose blood glucose remained higher than 180 mg/dl, 23.3% needed to be transferred to the ICU due to illness complications, compared to 4.5% of those whose glucose levels remained normal (p = 0.0001). The mortality rate in patients with hyperglycemia in the ICU was 16.1%, compared to 2.8% (p = 0.0001) among patients with normal glucose levels.

Although concurrent illness and medication changes may cause hyperglycemia during hospitalization, the low number of patients assessed for blood glucose made it impossible to assess the effects of hyperglycemia in a multivariate adjustment for all-cause mortality and illness complications associated with hyperglycemia.

Other investigators have previously found that even admission and persistent hyperglycemia events are associated with adverse outcomes and increased mortality after multivariate adjustment [13,19,23].

However, improved control has been shown to reduce mortality in several populations [7,8,28,29].

In this study, severe hypoglycemia, a complication that partially drives the undertreatment of hyperglycemia, was not as frequent as hyperglycemia. Hypoglycemia occurred in 7.2% of the patients in the ward and 24% in the ICU; hypoglycemia is avoidable with appropriate management.

Since the early 1990 s, it has been known [24,25] that the use of sliding-scale insulin in the absence of basal insulin is associated with wide glycemic variations. Consensus guidelines [4] and individual experts [26] suggest that optimal management of inpatient glycemia should include basal insulin with prandial insulin coverage rather than sliding scales alone.

In our analysis of data from one general hospital routine, sliding scales were prescribed as the sole treatment in 75% of the ward patients and 86% of the ICU patients, and insulin infusion was used only in 3.8% of the patients. Umpierrez et al. suggested that glycemic chaos (not glycemic control) is still the rule for inpatient care [26].

The restraints of using insulin infusion in patients in the ICU include a lack of inpatient diabetes management education for the hospital staff in addition to the absence of a treatment algorithm suited for the real-life hospital setting in Brazil.

Another limitation of our study was that we were not able to determine the percentage of patients with latent or unrecognized diabetes because of the lack of hemoglobin A1C testing. The prevalence of elevated hemoglobin A1C among patients admitted to the hospital without a diagnosis of diabetes was high [27]. Green et al. showed in a prospective cohort analysis of patients with diabetes and acute coronary syndrome that almost a third of the patients did not have an HbA1C value checked by the time of discharge.

Management of hyperglycemia in the hospital setting includes the measurement of HbA1C [20], and newly diagnosed inpatients represent an opportunity to institute a plan for long-term glycemic control. If initiated early, such intervention may lead to the prevention of complications [30].

Unfortunately, patients with newly noted hyperglycemia and established diabetes are frequently ignored in the hospital [4].

Finally, because of data limitations, this study was unable to test the hypothesis that hyperglycemia was the cause for the worst outcomes in patients hospitalized. Prior studies showed that hyperglycemic patients had higher in-hospital mortality and morbidity rates than those with normal glucose levels [1,4,7,14,15].

This analysis of a general hospital in Brazil revealed persistent shortcomings in inpatient hyperglycemia management. Inpatient diabetes care delivery may require systematic changes to meet current standards.

## Conclusions

In conclusion, we observed that hyperglycemia is common in a hospital setting and is associated with increased mortality and morbidity and longer length of hospital stay. Thus, it is necessary to review the in-hospital management of hyperglycemia by establishing an adequate protocol and continuous education and training within the hospital setting for providers, nurses, and ancillary staff.

## Competing interests

The authors declare that they have no competing interests.

## Authors' contributions

SAOL participated in the design of the study, write the manuscript and is the Senior of the project. SBL participated in the design of the study and participated in the collect data, SPN participated in the collect data, ARFO participated in the design of the study and participated in the collect data, DT participated in the design of the study and participated in the collect data, TT participated in the design of the study and participated in the collect data. All authors read and approved the final manuscript.
